# *LEF1* Induces *DHRS2* Gene Expression in Human Acute Leukemia Jurkat T-Cells

**DOI:** 10.4274/tjh.galenos.2020.2020.0144

**Published:** 2020-11-19

**Authors:** Sema Sırma Ekmekci, Zeliha Emrence, Neslihan Abacı, Melda Sarıman, Burcu Salman, Cumhur Gökhan Ekmekci, Çağrı Güleç

**Affiliations:** 1 İstanbul University, Aziz Sancar Institute of Experimental Medicine, Department of Genetics, İstanbul, Turkey

**Keywords:** T-cell acute lymphoblastic leukemia, p53, DHRS2, LEF1, siRNA

## Abstract

**Objective:**

T-cell acute lymphoblastic leukemia (T-ALL) is an aggressive disease resulting from the accumulation of genetic changes that affect the development of T-cells. The precise role of lymphoid enhancer-binding factor 1 (*LEF1*) in T-ALL has been controversial since both overexpression and inactivating *LEF1* mutations have been reported to date. Here, we investigate the potential gene targets of *LEF1* in the Jurkat human T-cell leukemia cell line.

**Materials and Methods:**

We used small interfering RNA (siRNA) technology to knock down *LEF1* in Jurkat cells and then compared the gene expression levels in the *LEF1* knockdown cells with nontargeting siRNA-transfected and non-transfected cells by employing microarray analysis.

**Results:**

We identified *DHRS2*, a tumor suppressor gene, as the most significantly downregulated gene in *LEF1* knockdown cells, and we further confirmed its downregulation by real-time quantitative polymerase chain reaction (qRT-PCR) in mRNA and at protein level by western blotting.

**Conclusion:**

Our results revealed that *DHRS2* is positively regulated by *LEF1* in Jurkat cells, which indicates the capability of *LEF1* as a tumor suppressor and, together with previous reports, suggests that *LEF1* exhibits a regulatory role in T-ALL via not only its oncogenic targets but also tumor suppressor genes.

## Abstract (tr)

**Amaç:**

T-hücreli akut lenfoblastik lösemi (T-ALL), T-hücrelerinin gelişimini etkileyen genetik değişikliklerin birikmesinden kaynaklanan agresif bir hastalıktır. Bugüne kadar lymphoid enhancer-binding factor 1’in (*LEF1*) hem inaktive edici mutasyonları hem de aşırı ekspresyonu bildirildiğinden T-ALL gelişiminde rolü tartışmalıdır. Bu çalışmada, insan T hücreli lösemi hücre soyu olan Jurkat hücrelerinde *LEF1*’in potansiyel hedef genleri araştırıldı.

**Gereç ve Yöntemler:**

Jurkat hücrelerinde küçük engelleyici RNA (siRNA) teknolojisi ile *LEF1* baskılandı ve mikroarray analizi ile *LEF1* baskılanmış hücrelerdeki gen ekspresyon seviyeleri, negatif kontrol siRNA (hedefsiz siRNA) ile transfekte edilmiş hücrelerdeki gen ekspresyon seviyeleri ile karşılaştırıldı.

**Bulgular:**

*LEF1* baskılanmış hücrelerde en önemli seviyede expresyonu azalan gen tümör baskılayıcı *DHRS2* geni olarak belirlendi. Ayrıca bu bulgu hem mRNA seviyesinde gerçek zamanlı kantitatif polimeraz zincir reaksiyonu (qRT-PCR) ile hem de protein seviyesinde western blot ile doğrulandı.

**Sonuç:**

Bu bulgular *DHRS2*’nin Jurkat hücrelerinde *LEF1* tarafından pozitif olarak düzenlendiğini göstererek *LEF1*’in tümör baskılayıcı kapasitesi olduğuna işaret etmektedir ve daha önceki çalışmalarda bildirilen sonuçlarla birlikte, *LEF1*’in hem onkojenik hedefleri hem de tümör baskılayıcı hedefleri aracılığıyla T-ALL’de düzenleyici bir rol oynadığını ileri sürmektedir.

## Introduction

T-cell acute lymphoblastic leukemia (T-ALL) is a malignancy associated with a significant risk of relapse and poor prognosis [1]. T-ALL represents approximately 25% of adult and 15% of pediatric ALL patients [2]. Although the prognosis of T-ALL has gradually improved over the years due to modern treatment protocols, resistance and relapse still remain major challenges in treatment. Thus, our understanding of molecular pathogenesis and the classification of patients can improve treatment outcomes and thereby increase success rates [3,4]. Activating mutations in *NOTCH1* or inactivating mutations in its negative regulator (*FBXW7*) occur in about 60% of T-ALL cases [5,6,7,8]. Activation of the *NOTCH* signaling pathway cooperates with loss of *p16/INK4A* and *p14/ARF*. In addition, translocations in oncogenes, such as LIM-only domain (*LMO*) genes, homeobox (*HOX*) genes, *MYC*, and *MYB*, frequently place these genes under the control of strong T cell-specific enhancers, thus causing aberrant overexpression [2,5].

Lymphoid enhancer-binding factor 1 (*LEF1*), a downstream transcriptional regulator of the Wnt/β-catenin pathway, regulates many cell cycle regulatory and cellular proliferation genes [9]. *LEF1* can also modulate gene transcription independently [10]. Previous studies have shown that *LEF1* plays a crucial role in normal hematopoiesis [9,11]. Defective pro-B cell survival and proliferation have been shown in *LEF1* knockout mice. Overexpression of *LEF1* in bone marrow progenitors results in B-lymphoblastic and acute myeloid lymphoma in recipient animals [11]. In leukemia and solid tumors, abnormal changes in *LEF1* expression have been reported in several studies [12,13,14,15].

The findings on the prognostic significance of *LEF1* expression show inconsistency among previously reported studies. For example, *LEF1 *expression has been found to be associated with poor prognosis in adult precursor B-cell acute lymphoblastic leukemia and chronic lymphocytic leukemia [14,16,17], while overexpression of *LEF1* has been determined as a favorable prognostic factor in childhood ALL and acute myeloid leukemia [13,18,19,20].

Many gene targets of *LEF1* and their associated pathways have been identified. However, its precise role in T-ALL has not been clarified yet. While some studies have shown an increased expression of *LEF1* in both premalignant thymocytes and T-ALL [16], others have reported the deletion of the *LEF1* gene accompanied with *NOTCH1* and *PTEN* mutations, biallelic *INK4A/ARF *(*CDKN2A*) deletions, or activating *PI3K* or *AKT* gene mutations in T-ALL [16,21,22]. These contradictory findings necessitate further studies to understand the molecular mechanism of *LEF1* in T-ALL.

In this study, we have investigated *LEF1*-regulated genes in Jurkat, a well-characterized human T acute lymphoblastic leukemia cell line that is widely used in a variety of studies to understand T-cell biology and T-cell signaling. The aim of our study was to identify potentially critical *LEF1*-regulated genes as well as related molecular signaling pathways using the Jurkat line as model cells.

## Materials and Methods

### Cell Culture

Jurkat cells were cultured at 37 °C with 5% CO_2_ in RPMI-1640 medium (Lonza, Basel, Switzerland) containing 10% fetal bovine serum (Capricorn Scientific, Ebsdorfergrund, Germany), 100 U/mL penicillin, 100 mg/mL streptomycin (GIBCO, Thermo Fisher Scientific, Waltham, MA, USA) and 2 mM L-glutamine.

### *LEF1* Small Interfering RNA (siRNA) Transfection

Jurkat cells were transfected with 100 nM *LEF1 *siRNA (SMARTpool ON-TARGET plus siRNA, Dharmacon, Lafayette, CO, USA), which targets both long (transcript variant 1, NCBI ID: NM_016269.5) and short isoforms (transcript variants 2, 3, 4; NCBI IDs: NM_001130713.2, NM_001130714.2, NM_001166119.1, respectively) of *LEF1 *or 100 nM non-targeting siRNA (SMARTpool ON-TARGET plus siRNA, Dharmacon) with HiPerFect transfection reagent (QIAGEN GmbH, Hilden, Germany) according to the manufacturer’s protocol and cultured for 24 and 48 h.

### RNA Isolation

Total RNA was isolated from Jurkat cells using the RNeasy Mini Kit (QIAGEN) in accordance with the manufacturer’s instructions. RNA concentrations were measured using a spectrophotometer (NanoDrop ND-1000, Thermo Scientific, Waltham, MA, USA).

### Real-Time Quantitative PCR (qRT-PCR)

*LEF1* siRNA knockdown and microarray results were confirmed by qRT-PCR. Reverse transcription was performed using random hexamers, total RNA, and the Transcriptor First Strand cDNA Synthesis Kit (Roche Life Science, Mannheim, Germany) following the manufacturer’s manual. To quantify the gene expression, primers specific to the *LEF1 *gene,* DHRS2* gene, and housekeeping *TATA binding protein *gene (*TBP*) were designed. qRT-PCR was performed using LightCycler 480 SYBR Green I Mix (Roche) and LightCycler 480 Instrument II (Roche) under the following PCR conditions:  95 °C for 5 min, 95 °C for 20 s, 64 °C for 20 s, and 72 °C for 15 s (45 cycles). Forward and reverse primers (5’-3’) were as follows: *TBP*-forward: ACT TGA CCT AAA GAC CAT TGC AC and *TBP*-reverse: CTT GAA GTC CAA GAA CTT AGC TGG; *DHRS2-*forward: CGA CTT CCT GGT GTG CAG and *DHRS2-*reverse: GTT CTC CAT GTA GGG CAG C; *LEF1*-forward TGG TGC AGC CAT CCC ATG and *LEF1*-reverse CGT GAT GGG ATA TAC AGG CTG ACC. Quantification was performed using the relative standard curve method. Each experiment was performed in triplicate. Gene expressions were normalized using the housekeeping gene *TBP*.

### Microarray

Microarray experiments were performed using the Affymetrix GeneChip^®^ 3’ IVT Express Kit (Affymetrix, Santa Clara, CA, USA). Sample preparation was conducted in accordance with the manufacturer’s protocol.  Fragmented end-labeled cDNA was hybridized onto the Affymetrix GeneChip® HG-U133 Plus 2.0 Array according to Affymetrix’s standard procedure. After hybridization, the chip was stained and washed in the GeneChip Fluidics Station 450 (Affymetrix) and scanned by GeneChip Array Scanner 3000 G7 (Affymetrix). Expression signals were extracted and normalized using the Expression Console (Affymetrix), applying the robust multichip average (RMA) normalization method. The microarray expression data generated in this study are available in the NCBI Gene Expression Omnibus database (GEO; htt://www.ncbi.nlm.nih.gov/geo/) [23] under accession number GSE129917.

### Microarray Data Analysis

Differential gene expression analyses were performed using the *limma* package in R. One-way ANOVA was applied to the RMA expression values in order to determine whether genes were differentially expressed between three groups. Multiple-testing correction was applied to the p-values of the F-statistics to adjust the false discovery rate [24]. Expression level differences with p-values (FDR-corrected) of <0.05 and fold changes of  >2 were considered significant. Morpheus (https://software.broadinstitute.org/morpheus) was used for the heatmap visualization of gene expression level differences. The Database for Annotation, Visualization, and Integrated Discovery (DAVID) [25,26] web-based tool was used for the biological interpretation of differentially expressed genes. The identified genes were classified based on Gene Ontology Resource [27] annotations and associated pathways were determined using the Kyoto Encyclopedia of Genes and Genomes (KEGG) [28].

### Protein Isolation and Western Blotting

Western blotting was performed to detect *LEF1* and *DHRS2* protein expression in the cells. All protein samples were prepared from a pool of siRNA-treated culture cells (three wells), which were homogenized and treated with a RIPA lysis buffer system (Santa Cruz Biotechnology, Santa Cruz, CA, USA) on ice. β-Actin was used as an internal control. The protein concentrations were quantified using the Pierce BCA Protein Assay Kit (Thermo Fisher Scientific). A total of 15 µg of proteins were separated in 4%-12% Bis-Tris gels (Nupage Novex, Life Technologies, Bleiswijk, the Netherlands) and then transferred onto a nitrocellulose membrane using i-Blot Gel transfer stacks (Novex, Life Technologies). After incubation with blocking buffer (5% BSA) for 1 h at room temperature, western blotting was performed using primary antibodies against *p53* (dilution, 1:100, DO-1 sc126, Santa Cruz), *LEF1* (dilution, 1:250, sc8592, Santa Cruz), *DHRS2* (dilution, 1:200, abcam, ab83254), and β*-actin* (1:1000, I-19R sc1616K, Santa Cruz) by overnight incubation at 4 °C. After a washing step, the HRP-conjugated secondary goat anti-mouse antibody for *p53* (1:3,000, ab97023, abcam), rabbit anti-goat ab for *LEF1* (1:2,000 abcam, ab6741), goat anti-rabbit for β*-actin*, and *DHRS2 *(1:5,000, Abbkine A21020-1, Abbkine Scientific, Redlands, CA, USA) were added and incubated for 1 h at room temperature. Bands were visualized by the WesternBright Sirius system (Advansista, Menlo Park, CA, USA) and analyzed using an imaging system (Wealtec Keta, Wealtec Bioscience Co., Ltd., New Taipei City, Taiwan). For protein quantification, densitometric analyses were done using Image J software (http://rsbweb.nih.gov/ij/index.html).

### Statistical Analysis

SPSS 17.0 (SPSS Inc., Chicago, IL, USA) was used for data analyses. For both *LEF1* and *DHRS2*, mRNA expression level differences between study groups were assessed by Student’s t-test. Values of p<0.05 were considered statistically significant.

## Results

**Figure 1:**
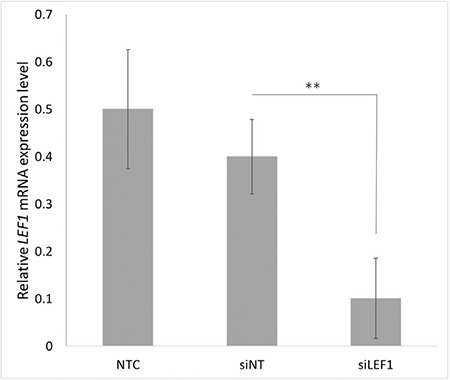
Expression of *LEF1* siRNA-transfected Jurkat cells by quantitative PCR. NTC: Non-transfected cells, siNT: non-targeting siRNA-transfected cells, si*LEF1*: *LEF1* siRNA-transfected cells. **: p=0.013.

In order to assess the efficiency of *LEF1* suppression after the transfection of Jurkat cells with *LEF1 *siRNA, we determined the mRNA levels of *LEF1* by real-time polymerase chain reaction (qRT-PCR). Twenty-four hours after transfection, we observed an approximately 74.7% reduction in *LEF1* siRNA-transfected (si*LEF1*) cells compared to non-targeting siRNA-transfected (siNT) cells (Figure 1).

**Figure 2:**
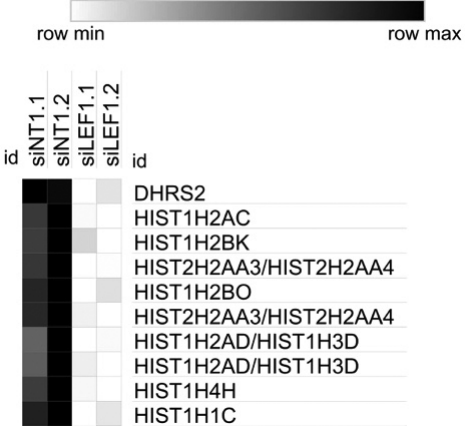
Heatmap of the most significant 10 DEGs. siNT: Non-targeting siRNA-transfected cells, si*LEF1*: *LEF1* siRNA-transfected cells.

We measured and compared gene expression levels between si*LEF1*, siNT, and non-transfected (NTC) Jurkat cells by microarray analysis, which revealed differentially expressed genes (DEGs), potential targets of *LEF1*. The most significant 10 DEGs included histone genes and *DHRS2* (Figure 2). The GO enrichment analysis of the significantly downregulated genes in si*LEF1* cells showed the distribution of the most abundant categories (Table 1). After GO enrichment analysis, we searched for the associated pathways for the DEGs using the KEGG and found that metabolic pathways, pathways in cancer, viral carcinogenesis, transcriptional dysregulation in cancer, mitogen-activated protein kinase signaling, and the *PI3K-Akt* pathway were among the aberrantly expressed signaling pathways in *LEF1*-downregulated cells (Table 2).

**Figure 3:**
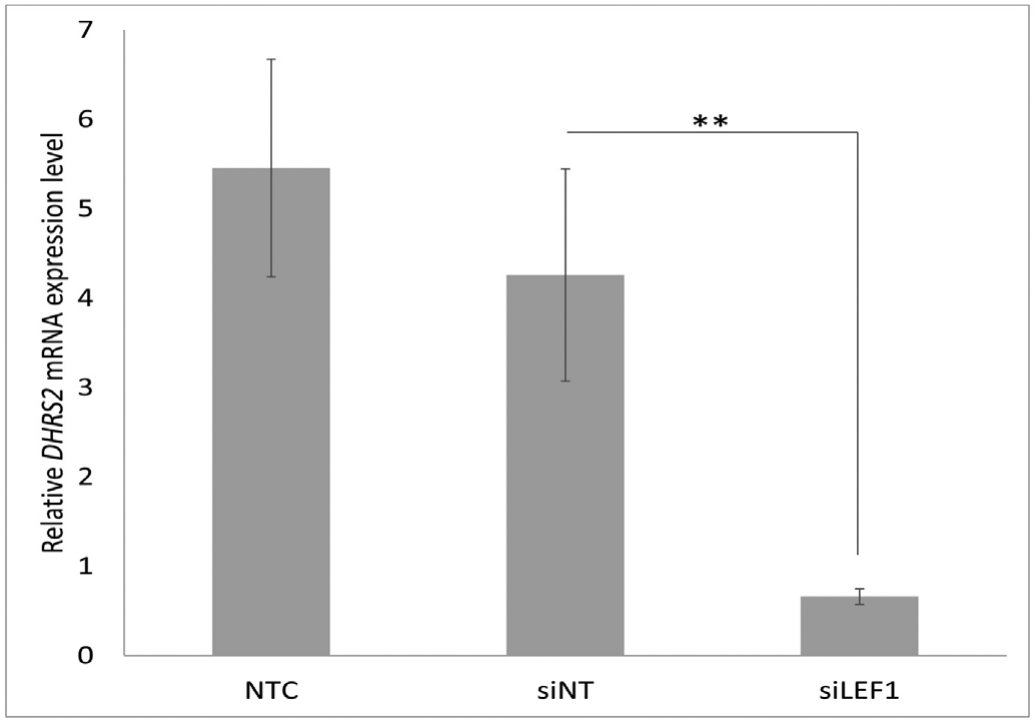
Expression of *DHRS2* siRNA-transfected Jurkat cells by quantitative polymerase chain reaction. NTC: Non-transfected cells, siNT: non-targeting siRNA-transfected cells, si*LEF1*: *LEF1* siRNA-transfected cells. **: p=0.001.

We verified our microarray results by comparison of *DHRS2* gene expressions among si*LEF1*, siNT, and NTC cells by qRT-PCR. Twenty-four hours after transfection, compared to siNT cells, an 84% decrease was observed in mRNA levels of *DHRS2* in si*LEF1* cells (Figure 3).

**Figure 4:**
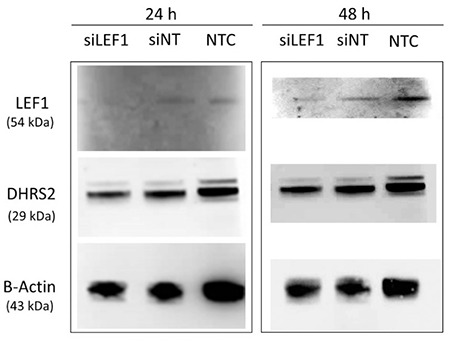
*LEF1* and *DHRS2* protein levels in si*LEF1*, siNT, and NTC cells 24 h and 48 h after transfection. NTC: Non-transfected cells, siNT: non-targeting siRNA-transfected cells, si*LEF1*: *LEF1* siRNA-transfected cells.

**Figure 5:**
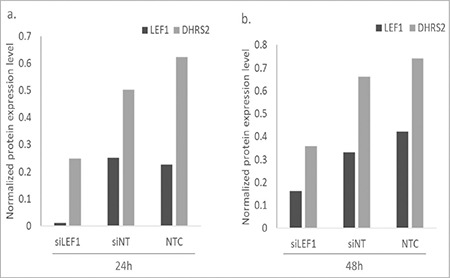
*LEF1* and *DHRS2* protein levels normalized by using β-actin protein expression level: a) 24 h after transfection, b) 48 h after transfection. NTC: Non-transfected cells, siNT: non-targeting siRNA-transfected cells, si*LEF1*: *LEF1* siRNA-transfected cells.

Protein level verification of microarray and qRT-PCR results was conducted by western blotting. Protein levels of *LEF1* and *DHRS2 *were determined to investigate the *LEF1* and *DHRS2* genes’ downregulation in si*LEF1* cells compared to siNT and NTC cells. *LEF1* protein levels were almost undetectable 24 h after transfection (Figure 4) and were reduced by 1.8-fold 48 h after transfection in si*LEF1 *cells compared to siNT cells (Figure 4). The protein level of DHRS2 was 2.1-fold reduced in si*LEF1* cells compared to siNT cells 24 h after transfection and the suppression persisted 48 h after transfection (Figure 4). *LEF1* and *DHRS2* protein levels obtained by western blotting were quantified by normalizing the protein expression levels to β*-actin* expression (Figure 5).

## Discussion

Although there have been many studies on T-ALL, the underlying molecular mechanisms of this disease have yet to be revealed. In this study, we examined the potential role of the transcription factor *LEF1* in T-ALL by determining its target genes and regulation mechanisms. We have compared the gene expression levels of si*LEF1*, siNT, and NTC Jurkat cells by microarray analysis in order to identify DEGs, which are potential targets of *LEF1* (https://www.ncbi.nlm.nih.gov/geo/query/acc.cgi?acc=GSE129917). One of the most enriched pathways for downregulated genes was “Pathways in cancer-hsa05200,” which is consistent with the association of *LEF1* expression with a variety of cancers. The most significant 10 DEGs included *DHRS2* (HEP27) and histone genes (Figure 2). As *LEF1* is known to regulate cell cycle regulators and cellular proliferation genes, the accompanying downregulation of histone genes in *LEF1* knockdown cells reflects the relationship between *LEF1* and cellular proliferation. We further focused on *DHRS2*, which is a member of the short-chain dehydrogenase/reductase enzyme family that has activity toward steroids, retinoids, prostaglandins, and xenobiotics [29,30]. Thus, to verify our microarray results, we analyzed the expression levels of *LEF1* and *DHRS2* in si*LEF1*, siNT, and NTC cells using qRT-PCR. Additionally, protein levels of these two genes were evaluated by western blotting. Both RNA and protein level analyses confirmed our microarray results. We also searched the GEO database and found that the *DHRS2* gene is upregulated in colon cancer cells treated with the adenoviral *LEF1* expression vector (GEO accession number: GSE3229), which is consistent with our results.

*DHRS2* is suggested to be a tumor suppressor gene in different tumor types, including nasopharyngeal carcinoma [31,32], gastrointestinal stromal tumors [33,34], metastatic lung adenocarcinomas [35], esophageal squamous cell carcinoma [30], and renal cancer [36]. Previous reports showed that the *DHRS2* enzyme interacts with *MDM2*, a protein responsible for the negative regulation of the *p53* tumor suppressor gene [37,38,39]. Similarly, it is also known that one of the alternatively spliced transcripts of *CDKN2 *(*ARF*) antagonizes *MDM2*-dependent p53 degradation [40]. Furthermore, *LEF1* inactivation has been associated with biallelic *INK4a/ARF* deletions in T-ALL [21]. Additionally, it has been reported that overexpression of β-catenin, a coactivator of *LEF1*, results in p53 accumulation through upregulation of *ARF* [41,42] and the N-terminal of *LEF1* (ΔNLef1), which acts as a tumor promoter by preventing accumulation of p53 in human and mouse sebaceous tumors, and *ARF* downregulation is likely to be responsible for this mechanism [43]. Thus, it may be possible that the activation of p53 accumulation by β-catenin and *LEF1* depends on not only *ARF* but also *DHRS2* upregulation. However, further functional studies are needed to investigate these relationships and understand the molecular mechanism.

*p53* mutations are known to be frequent in T-ALL [44,45]. In Jurkat cells, a heterozygous, stop-gained mutation in exon 6 of the *p53* gene (R196* or rs397516435) considered to be important in leukemogenesis or in the tumorigenic progression of leukemic T cells has been reported [46]. Thus, as Jurkat cells are *p53-*mutant, we could not detect p53 in western blotting analysis. Our findings imply that *DHRS2*-mediated p53 accumulation does not occur in* p53*-mutant Jurkat cells and overexpression of *LEF1 *may show oncogenic effects via overexpression of its downstream target, *MYC*, which is known to play a major role in T-ALL [6,47]. It has been reported that *LEF1* is overexpressed in 30% of adult T-ALL patients [16]. On the other hand, *LEF1* microdeletion was detected in 11% of adult T-ALL cases [21]. These contradictory observations might result from the altered *LEF1* effects due to cooperative tumorigenic genetic events. It is known that both oncogenes and tumor suppressor genes are targeted by *LEF1*, which suggests that cooperative genetic events in its downstream genes may determine the final outcome of *LEF1* action. Our results suggest that *DHRS2* is one of the tumor suppressor targets of *LEF1* in the Jurkat human T-cell leukemia cell line. Based on these results, one may speculate that the inactivation of *LEF1* may be causing the prevention of the tumor suppressor effect of *DHRS2* in T cells and contributing to leukemogenesis.

## Conclusion

In this study, we demonstrate that *LEF1* positively regulates *DHRS2* gene expression in the Jurkat human T-cell leukemia cell line and thus provide new insight into the *LEF1-p53* link in T-cell leukemogenesis. Our findings suggest a tumor-suppressive role for *LEF1* by the regulation of the downstream *DHRS2*-*p53* signaling pathway, which explains the molecular mechanism behind the observation of *LEF1*-induced p53 accumulation. This study supports the growing evidence that *LEF1* plays a regulatory role in T-cell proliferation and differentiation and its dysregulation contributes to the development of T-ALL. The main limitations of our study are that it was performed by using only one cell line, was not validated in T-ALL patients, and requires further functional investigations to confirm the implications of its results, including the potential role of *DHRS2* in T-ALL and its interactions with *LEF1*.

## Figures and Tables

**Table 1:**
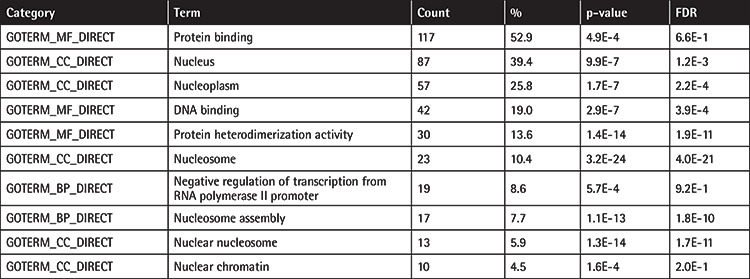
Top 10 most enriched GO terms for downregulated genes in *LEF1* knockdown cells.

**Table 2:**
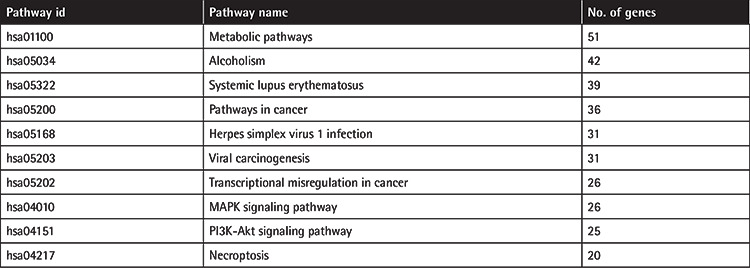
Top 10 KEGG pathways according to the number of associated DEGs.
